# Remission in Depression and Associated Factors at Different Assessment Times in Primary Care in Chile

**DOI:** 10.2174/1745017901814010078

**Published:** 2018-03-26

**Authors:** Veronica Vitriol, Alfredo Cancino, Carlos Serrano, Soledad Ballesteros, Soledad Potthoff

**Affiliations:** 1 Medicine School, Universidad de Talca, Talca, Chile; 2 Psychology School, Universidad de Talca, Talca, Chile; 3 Neurosciences Institute, Viña del Mar, Chile

**Keywords:** Depression, Remission, Primary health care, Child abuse, Logistic, Dichotomous

## Abstract

**Objective:**

To determine the factors associated with remission at 3, 6, 9 and 12 months among depressive adult patients in primary care [PHC] in Chile.

**Methods:**

This is a one-year naturalistic study that followed 297 patients admitted for treatment of depression in eight primary care clinics in Chile. Initially, patients were evaluated using: the International Mini-Neuropsychiatric Interview [MINI], a screening for Childhood Trauma Events [CTEs], the Life Experiences Survey and a partner violence scale. The Hamilton Depression Scale [HDRS] was used to follow the patients during the observation time. Associations between the factors studied and the primary outcome remission [HDRS ≤ 7] were assessed using a dichotomous logistic regression and a multivariate Poisson regression. The significance level was 0.05.

**Results:**

Remission [HDRS ≤ 7] ranged between 36.7% at 3 months and 53.9% at 12 months. Factors that predicted poor remission during the observation time were: CTEs [Wald X^2^ = 4.88, Exp B=0.94, CI 0.90-0.92, p=0.27]; psychiatric comorbidities [Wald X^2^ = 10.73, Exp B=0.90, CI 0.85-0.96, p=0.01]; suicidal tendencies [Wald X^2^ = 4.66, Exp B=0.88, CI 0.79-0.98, p=0.03] and prior treatment for depression [Wald X^2^ = 4.50, Exp B=0.81, CI 0.68-0.85, p=0.03]

**Discussion:**

Almost 50% of this sample failed remission in depression at 12 months. Psychiatric comorbidities and CTEs are factors that should be considered for a poor outcome in depressed Chilean patients. These factors need more recognition and a better approach in PHC.

## 1 INTRODUCTION

In Chile, as in the world, major depression is a relevant public health problem [1, 2]. Among the general Chilean adult population, it is estimated that lifetime prevalence of major depressive episodes is 9% [3] and the prevalence of depressive symptoms during the last year reached 17.2% [4].

Since 2001, in the Chilean health system, a specific program to treat depression has been implemented [5]. This program guarantees care and treatment costs according to the recommendations of the national depression guidelines [6].

The current clinical guide provides recommendations for detection, diagnosis and treatment for depression at different levels of public health care. This guide categorizes the severity of depression based on the number of depressive symptoms, according to the tenth version of the International Classification of Diseases (ICD-10) [6]. Mild, moderate and severe depressions without a current suicide attempt are treated in primary care (PHC). Only depressed patients with a current suicide attempt, psychosis, bipolarity and/or therapeutic refractoriness are sent from PHC to specialized treatment [6].

In Chile, 90% of depression cases are solved by General Practitioners (GPs) and psychosocial teams in PHC [5, 6]. However, despite 15 years of the development of the national depression program [4, 5], there is still no evidence on the natural course of this illness and the factors associated with a worse outcome in PHC. To promote evidence-based decisions, current knowledge in this area is required.

According to the current knowledge, the aim of depression treatment in PHC is the remission of depressive symptoms [7]. The evidence shows that this outcome reaches between 25% at 3 months and 50-70% at 12 months in PHC naturalistic studies carried out in North America, Europe and Asia [8-15]. In these studies, initial severity of depressive symptoms, psychiatric comorbidities, biomedical conditions and poor adherence to treatment are the factors most associated with poor remission [10-15].

There is a lack of evidence in PHC regarding the factors that are associated with a worse outcome and the time when the same sample is evaluated. Understanding this could contribute to the recognition of subgroups of PHC depressed patients who could achieve remission at different times, according to clinical or psychosocial factors present at baseline.

Another issue that needs more evidence in PHC is the influence of Childhood Trauma Events (CTEs) and other adverse biographical experiences on the natural evolution of depression.

According to studies developed during the last thirty years [16, 17], there is enough evidence showing a preeminent role of CTEs in vulnerability for developing major psychiatric disorders such as depression [16, 17]. This vulnerability is an expression of altered stress responsiveness and a consequence of neurobiological alterations in the circuitry involved in stress and emotion regulation [16].

Depressed patients with a history of childhood abuse present a complex clinical picture, characterized mainly by the presence of greater comorbidity, chronicity, suicidality, the occurrence of other adverse events during adulthood and unresponsiveness to pharmacological treatments [18-28]

However, despite the deleterious consequences of CTEs in adult health, these backgrounds are not properly inquired in adult subjects when they consult health services [29]. Probably, in relation to this fact, there is a lack of evidence regarding the influence of CTEs on depression remission in PHC.

Specifically in Chile, there is evidence that shows that between 60 - 80% of depressed patients report having been exposed to at least one CTE during their life [30], associated with more severity and comorbidity of depression [30, 31]. In addition, almost half of the women diagnosed with depression in PHC reported having been victims of violence inflicted by their partners [32].

This research aimed to determine remission rates at 3, 6, 9 and 12 months, in a sample of depressed patients in Chilean PHC, and, in turn, to establish which factors predict poor remission at each time of evaluation and which could have an effect on the number of remissions over the observation period.

## 2 METHODS

### 2.1 Design

A longitudinal cohort study was conducted in the Maule Region in Chile between February and September 2014. The protocol was approved by the Ethics Committees of the Universidad de Talca and the Maule Regional Health Service. All subjects provided verbal and written informed consent prior to participation.

### 2.2 Sample

 Considering previous national studies, a convenience sample was calculated with 95% of confidence, 80% of power and a 30% dropout rate. According to this, 336 cases were needed.

The cases were recruited from 2984 patients older than 15 years that were admitted to PHC treatment for depression in 8 urban clinics between February and September 2014. Patients were invited by their GPs to participate in this study. In turn, they also determined in each patient the autonomy to sign an informed consent.

Exclusion criteria included organic brain damage, sensory disability, referral to secondary care at the time of admission due to current severe suicide attempt, bipolarity and/or psychosis.

When 440 patients had been evaluated by the research team, the recruitment time ended.

Based on the ICD-10 criteria [33], a depressive episode diagnosis was confirmed by psychiatrists and psychologists using the Mini-International Neuropsychiatric Interview (MINI) [34]. Forty-six patients did not meet the criteria for a depressive episode. Thus, 394 patients were admitted in the study.

### 2.3 Instruments

At baseline, all the participants were interviewed by the specialized team using the following instruments:

1- Screening for CTEs [35], evaluating whether the patients remember having suffered one of the following events before the age of 15: traumatic separation from caregiver during more than one month, alcohol or drug abuse by a family member, physical abuse, physical injury associated with the physical abuse, domestic violence between parents or caregivers, and sexual abuse by a relative and/or non-relative. This instrument has been validated and used in Chile in previous works [36-38].
2- Life Experiences Survey (LES). It consists of 47 items that investigate vital changes that occurred during previous six months with both positive and negative connotations [39]. A Spanish translation [40] was used. For the purposes of this study, only the negative life experiences were considered.
3- Questionnaire for Intimate Partner Violence (IPV). This instrument consists of 12 questions based on the World Health Organization (WHO) definition for physical, psychological and sexual violence, with frequencies of never, once or twice, and three or more times. This questionnaire has been used in previous studies in Chile [41].
4- Mini International Neuropsychiatric Interview (MINI) [34]. It is a brief and highly structured interview that investigates major psychiatric disorders listed in the ICD-10 and in the fourth edition of the Diagnostic and Statistical Manual of Mental Disorders (DSM-IV).
5- Hamilton Rating Scale for Depression (HDRS). This 17 item version was used to determine the severity of the disease at baseline [42]. This scale was also used to follow-up the patients at 3, 6, 9, and 12 months. A score ≤ 7 was considered indicative of remission [43].

Longitudinal data collection occurred between April 2014 and October 2015.

### 2.4 Statistical Data Analysis

The statistical data analysis was conducted using the fourteenth version of the Statistical Package for the Social Sciences (SPSS) program.

A quality control of consistency of data was performed at the beginning and at each of the periods in order to ensure the reliability of data and the patient's response. The specialized team made sure to complete all the initial assessment instruments.

In the case of patients with one missing data item or two non-consecutive missing data items in the HDRS, their measurements were considered through a simple imputation.

A dichotomous logistic regression analysis was performed in order to determine the factors that explained lower remission of depressive symptoms at 3, 6, 9 and 12 months. Finally, a multivariate Poisson regression analysis to determine the factors that had an effect on the number of remissions during observation time was performed. The significance level was 0.05.

## 3 RESULTS

### 3.1 Sample Characteristics Table **1**

Most patients were women (86.8%), who consulted during middle age (average 47.5 years) and a half lived with a partner and without paid activity. Near half of patients reported having received previous treatment for depression and one suicide attempt was reported by 35% of participants.

As for comorbidities, at least a biomedical pathology was presented in 55% of the patients and 89.1% presented one comorbid psychiatric disorder.

Regarding biographical adversity, 82% of participants reported having been exposed to at least one CTE, 58.1% reported some type of IPV in their lifetime and 92.4% reported having experienced one or more stressful vital event during last 6 months.

### 3.2 Remission Rates and Associated Factors

Regarding the initial sample, at 3 months were evaluated 309 patients (78.4%), at 6 months 344 patients (87%), at 9 months 256 patients (64.9%) and at 12 month 289 patients (73.3%).

On the other hand, 204 patients completed all assessments, 35 participants missed only one evaluation and 58 patients missed two non-consecutive evaluations. The final cohort sample included 297 patients.

Remission rates observed in the final cohort size sample were: 36.7% at 3 months; 47.8% at 6 months; 52.2% at 9 months and 53.9% at 12 months.

Table **2** shows the factors that were associated with lack of remission at each time of evaluation. At 3 months: older age (Exp B=1.02, CI 1.0 -1.05, p=0.05) CTEs (ExpB =1.25, CI 1.07-145, p=0.004) and psychiatric comorbidity [Exp B=1.34, CI 1.1-1.32, p=0.03]. At 6 months: previous depressive episodes (Exp B=1.07, CI 0.99 -1.14, p=0.05) at 9 months: psychiatric comorbidity (Exp B 1.4, CI 1.17 -1.68, p= 0.00) and at 12 months: suicide tendencies (Exp B=1.26, CI 1.02 -1.81, p=0.03)

Finally, according to the multivariate Poisson regression, the Chi square test indicates that the proposed model allows a significant reduction in the size of the misfit (X^2^ = 40.98; p = 0.00]. In this case, the variables that were significant for the prediction of total remissions during the evaluation time were: CTEs [wald X^2^ = 4.88, Exp B=0.94, CI 0.90-0.92, p=0.27); psychiatric comorbidities (wald X^2^ = 10.73, Exp B=0.90, CI 0.85-0.96, p=0.01); suicidal tendencies (wald X^2^ = 4.66, Exp B=0.88, CI 0.79-0.98, p=0.031) and prior treatment for depression (wald X^2^ = 4.50, Exp B=0.81, CI 0.68-0.85, p=0.03) (Table **3**).

Including these four variables, the model can be improved [X2 = 187.75; p = 0.00] and in this case near 3 or near 4 patients of the sample remit when there are no history of CTEs or psychiatric comorbidities or history of prior treatment for depression. (Exp B = 3.18, CI 2.69 3.76, p = 0.00) (Table **4**).

## 4 DISCUSSION

In Chile, this is the first naturalistic study on the evolution of depressive symptoms in adult patients cared for at PHC, which could lead to a better understanding about their evolution and distinctive clinical features.

### 4.1 Remission Rates

Remission rates observed in this study were consistent with the findings of current literature [8-15]. In 2014, a meta-analysis of remission rates of undiagnosed depressed primary care patients estimated that about one-quarter of cases reported remission within 3 months, one third within 6 months and a half within 12 months [8]. In our study, the highest remission rate was observed at 3 and 6 months, which could be explained since this is a cohort of diagnosed depressed primary care patients.

On the other hand, Chin *et al*. [12] in a one-year naturalistic study conducted in patients with diagnosed and undiagnosed depression in Hong Kong PHC could not establish a relationship between a higher remission rate at 12 months and a greater recognition of depressive symptoms by GPs, and if this recognition was associated with a prior achievement of remission [12]. This evidence can explain the results in our study in which almost 50% of the patients remitted at six months, a figure that then increased very little in the year of observation.

It is important to note that the remission rates observed in our sample were higher than those observed in the depressive samples treated at a specialized level in Chile. According to the national clinical guide, depressed patients with current suicide attempt, psychosis, bipolarity and/or therapeutic refractoriness are sent from PHC to specialized treatment [4, 5]. In this country, according to two studies conducted at the specialized level, depression remission rates reach between 14% at six months and 27.5% at 12 months [38, 44].

Some authors propose that the difference between depression remission rates observed in PHC *versus* specialized care can be explained considering that the query in PHC is for mild depressive symptoms and/or adjustment disorders that could resolve spontaneously [8, 12]. In this line of thinking, we propose that a timely recognition of depressive symptoms by the PHC team (which in Chile has been optimized thanks to the implementation of a specific program) would favor the resolution of this symptomatology probably before the spontaneous remission.

Finally, it is important to note that almost 50% of patients of this sample failed to remit at 12 months. This result, which is consistent with the current literature [8-15], is associated with factors that can be recognized during the first consultation at PHC [45-47].

### 4.2 Factors Associated With Poor Remission

Through the analysis performed, history of childhood trauma, psychiatric comorbidity, suicide tendencies and previous depression are the factors that predicted a poor remission in this sample. These results are consistent with the evidence in depression literature [46].

Of all of these factors, emphasis is placed on the importance of CTEs. The research team found a high prevalence of CTE in this sample. This result supports the evidence from previous studies carried out in Chile [29], but it is higher than the reports of other depressed samples [48]. In addition, it is known that CTEs among depressed patients are associated with the same factors that were associated with lack of remission in our sample such as psychiatric comorbidities and suicidal tendencies [18, 21, 31].

Teicher *et al*. and other authors, on the evidence of the last 30 years, support the idea that the complex clinical picture presented in patients with depression who were maltreated during childhood represents the expression of a distinct endo-phenotype [16, 17]. This subtype of depression would need to be recognized and boarded in clinical practice through a different approach [49]. In this sense, the results of this study were consistent with and might be explained by the early trauma hypothesis. Moreover, these findings provide evidence that can guide the approach to this subtype of depression in PHC.

According to our data, the first analysis linked different factors to achieve remission in each evaluation time such as the CTEs that predicted poor remission at 3 months. This finding implies that depressed patients with a history of early trauma could need more time to achieve remission in PHC. However, considering the Poisson regression analysis, psychiatric comorbidities was the most important factor that predicted lack of remissions. Therefore, we propose that this factor should be prioritized, recognized and treated in patients with depression who consult in Chile PHC.

Regarding psychiatric comorbidity, the evidence indicates that anxiety disorders are underdiagnosed in depressed patients consulting in PHC [50]. This lack of recognition is associated with a worse prognosis of depression and suicide risk [50-52]. Moreover, patients with anxiety-depression comorbidity need a different pharmacological and psychological treatment [53]. The importance of anxiety in depression has been recognized and incorporated into the DSM V anxious subtype of depression [54].

The current clinical guideline in Chile offers recommendations based mainly on the recognition of depression symptoms according to ICD 10 [2, 5, 6]. Taking into account the high prevalence of anxiety disorders shown in this sample [30, 31] and their relationship to poor remission, it is suggested that GPs in Chile should be trained to recognize and manage these psychiatric comorbidities in depressed patients.

In contrast to what has been reported in several studies [9-11, 13, 55], in our sample as well as in the Hong Kong study [12], the greater initial severity of depression could not be associated with lack of remission. This [apparently contradictory] result could be explained considering the hypothesis previously exposed: at PHC, a subgroup of depressed patients present adjustment disorders with initial severe symptoms on the basis of a vulnerability derived from exposure to adverse biographical events since childhood. Among these patients, initial interventions in PHC or their own personal resilience could favor a rapid decline in depression.

Unlike what was expected, bio-medical comorbidity was not associated with lower remission in this sample. These results warrant further research, as the prevalence of medical conditions in depressed patients is two to three times higher than that reported in patients without depression [56] and medical comorbidity has been associated with a worse prognosis of depression as shown in other studies [57].

Finally, it is important to note that treatment for previous depression (which implies a chronic illness that already warranted a treatment) is a factor associated with a lower number of remissions during the observation time. This result may be associated to the same factors that predicted a worse prognosis in our sample. This requires more study and analysis since it could be a subgroup of patients who need a more-timely referral to the specialized level [46].

The main strengths of this research were conducting it in Latino patients and the quality of the data. In fact, during the year of observation, the attrition rate was low. In addition, the mental health team had access to highly confidential patient records and made sure that their assessments were carried out using reliable standardized instruments. Moreover, this study incorporated the analysis of several adverse biographical events since childhood.

We consider the following limitations: CTEs were investigated retrospectively and all psychiatric comorbidities or personality disorders were not inquired. The relationship between remission and recurrence during the year of observation was not analyzed. Furthermore, the treatment indicated and the use of health services should be studied and included in a future analysis. Finally, the data only came from the Maule Region and may not represent the reality of the rest of the country.

Despite the above limitations, these results provide further evidence about the evolution of depressed patients, studied in a naturalistic context in PHC according to a national program implemented during more than one decade. These results indicate that the prediction of a poor outcome in depression in PHC should not only be based on clinical features. Also, biographical adversity becomes an important factor that should be considered.

The clinical guidelines for the treatment of depression in Chile offer recommendations based mainly on the recognition of depressed symptoms according to ICD 10. On the other hand, it is known that neither biographical trauma nor psychiatric comorbidities are adequately inquired about when subjects consult for depression in PHC [28, 29, 50]. The findings of this study indicate that other strategies aimed at improving the recognition of these clinical and psychosocial factors in depressed patients should be incorporated in PHC.

## Figures and Tables

**Table 1:** Demographic, clinical and psychosocial characteristics of sample. 297 patients consulting for depression in primary care, Maule region, Chile 2017.

**DEMOGRAPHIC CHARACTERISTICS**		
**Age**	**Mean**	**SD**
	**47.5 years**	**15.1**
**Sex**	**N**	**%**
**Women**	**344**	**86.8**
**Scholarship**	**N**	**%**
**Without scholarship**	**5**	**1.5**
**Elementary not completed**	**98**	**24.8**
**Elementary completed**	**53**	**13.4**
**Middle not completed**	**54**	**13.7**
**Middle completed**	**110**	**27.9**
**High School not completed**	**30**	**7.8**
**High School completed**	**44**	**11.1**
**PSYCHOSOCIAL CHARACTERISTICS**	**N**	**%**
**Lives with partner**	**179**	**45.4**
**Lives alone**	**48**	**12**
**Incomes through gainful** **employement**	**52**	**13.2**
**CLINICAL CHARACTERISTICS OF DEPRESSION**		
–	**Mean**	**DS**
**Age of the first episode (years)**	**30.7**	**17.2**
**Number of depressive episodes**	**3.64**	**4.2**
**Duration of the longest episode (years)**	**3.58**	**7.22**
**Depressive symptoms at baseline HDRS (points)**	**20**	**4.6**
–	**N**	**%**
**History of suicide attempt**	**138**	**35**
**History of previous depression treatment**	**177**	**44.9**
**COMORBIDITIES**		
**Biomedical**	**N**	**%**
0	177	45
1	**84**	**21.4**
3	74	18,9
**More than 3**	59	15
**Psychiatric**	**N**	**%**
**0**	**43**	**10,9**
**1**	**81**	**20.6**
**2**	**80**	**20.3**
**3**	**77**	**19.5**
**More than 3**	**113**	**28.6**
**BIOGRAPHICAL STRESSFUL EVENTS**		
**Life experiences during previous six months**	**N**	**%**
**0**	**30**	**7.6**
**1**	**83**	**21.1**
**2**	**87**	**22.1**
3	**64**	**16.2**
**More than 3**	**136**	**34.6**
**Intimate partner violence events (entire life)**	**N**	**%**
**0**	**165**	**41.9**
**1**	**25**	**6.4%**
**2**	**18**	** 4.7%**
**3 or more**	**186**	**47.2%**
**Childhood trauma events**	**N**	**%**
**0**	**71**	**18**
**1**	**67**	**17**
**2**	**66**	**16.7**
**3**	**67**	**17**
**4 or more**	**123**	**31.2**

**Table 2:** Factors associated with lack of remission in different times of assesment. 297 patients consulting for depression in primary care, Maule region, Chile 2017.

**Variables**	**3 MONTHS**			**6 Months**			**9 Months**			**12 Months**		
–	**Exp(B)**	**CI 95%**	**Sig.**	**Exp(B)**	**CI 95%**	**Sig.**	**Exp(B)**	**CI 95%**	**Sig.**	**Exp(B)**	**CI 95%**	**Sig.**
**Gender**	1.025	.467-2.251	.951	1.513	.696-3.286	.296	1.091	.493-2.414	.829	1.089	.495-2.398	.832
**Education**	1.101	.932- 1.301	.257	.991	.850-1.156	.909	1.031	.879-1.208	.709	1.037	.888-1.211	.644
**Older Age**	**1.025**	**1-1.05**	.05*	1.002	.979-1.025	.877	1.007	.983-1.031	.578	1.007	.985-1.030	.531
**Employment**	1.225	.705-2.131	.472	1.535	.913-2.581	.106	1.259	.739-2.145	.397	1.227	.730-2.063	.440
**Age of onset depression**	.992	.976-1.009	.371	1.006	.990-1.022	.461	1.009	.993-1.026	.282	1.004	.988-1.020	.626
**Number of depressive episodes**	.989	.922-1.061	.754	**1.07**	**1.0-1.15**	0.05*	1.055	.985-1.131	.128	.998	.937-1.064	.961
**Suicidal tendencies**	1.257	.924-1.710	.145	.911	.687-1.206	.513	1.308	.980-1.745	.068	**1.37**	**1.03-1.8**	.031 *
**Childhood trauma events**	**1.25**	**1.07-1.45**	.004 ***	1.082	.943-1.242	.260	1.089	.946-1.254	.235	1.107	.965-1.270	.147
**Adverse life stressful events prior 6 months**	.916	.804-1.043	.184	.928	.822-1.046	.220	.911	.804-1.032	.144	.920	.816-1.038	.176
**Partner violence**	1.012	.941-1.088	.749	.972	.910-1,039	.407	.996	.930-1.067	.915	1.017	.952-1.087	.611
**Psychiatric comorbidities**	**1.34**	**1.1-1.62**	.003 ***	1.110	.946-1.302	.201	**1.4**	**1.17-1.68**	**.000**	1.147	.972-1.353	.104
**Medical comorbidities**	1.044	.815-1.339	.731	1.125	312-1.414	.895	.908	.720-1.146	.416	1.114	.889-1.396	.348
**Hamilton basal severity**	1.108	.868-1.415	.409	1.079	.519-1.359	.856	1.156	.911-1.466	.232	1.110	.878-1.402	.383
**Treatment for prior depression**	1.342	.757-2.382	.314	1.418	.195-2.404	.836	1.219	.706-2.104	.478	1.566	.918-2.670	.100

*p<0.05 ** p<0.01 ***p<0.001

**Table 3:** Parameters poisson regression in remission counts, 297 patients consulting for depression in primary care, Maule region, Chile 2017.

**Parameters Estimation**																
Parameter	B	Error type		Wald confidence interval 95%				Hypothesis testing				Exp(B)	Wald Confidence interval of Exp(B) 95%			
				Lower		Higher		Wald x^2^		Gl	Sig.		Lower		Higher	
(Intersection)	1.538	.2402		1.067		2.009		40.987		1	.000	4.655	2.907		7.455	
Employement	-.105	.0916		-.284		.075		1.309		1	.253	.901	.753		1.078	
Age	-.005	.0033		-.011		.002		1.980		1	.159	.995	.989		1.002	
Gender	-.040	.1289		-.292		.213		.095		1	.757	.961	.746		1.237	
Number kids	.010	.0286		-.046		.067		.132		1	.716	1.010	.955		1.069	
Education level	-.006	.0280		-.061		.049		.049		1	.825	.994	.941		1.050	
Trauma childhood events	**-.055**	**.0248**		**-.103**		**-.006**		**4.883**		**1**	**.027**	**.947**	**.902**		**.994**	
Partner Violence events	.001	.0120		-.023		.024		.003		1	.956	1.001	.977		1.025	
Suicidal tendencies	**-.123**	**.0571**		**-.235**		**-.011**		**4.657**		**1**	**.031**	**.884**	**.790**		**.989**	
Life stressful events prior 6 months	.032	.0211		.009		.074		2.346		1	.126	1.033	.991		1.077	
Psychiatric Comorbidities	**-.102**	**.0312**		**-.163**		**-.041**		**10.733**		**1**	**.001**	**.903**	**.849**		**.960**	
Biomedical comorbidities	-.016	.0436		-.102		.069		.136		1	.712	.984	.903		1.072	
Treatment for previous depression	**-.201**	**.0947**		**-.387**		**-.015**		**4.507**		**1**	**.034**	**.818**	**.679**		**.985**	
Inicial Hamilton severity	-.068	.0408		-.148		.012		2.783		1	.095	.934	.862		1.012	

**Table 4:** Parameters poisson regression in remission counts, improved model, 297 patients consulting for depression in primary care, Maule region, Chile 2017.

Parameter	B	Error type		Wald confidence interval 95%				Hypothesis testing				Exp(B)	Wald Confidence interval of Exp(B) 95%			
				Lower		Higher		Wald x^2^		Gl	Sig.		Lower		Higher	
(Intersection)	1.159	.0845		.993		1.324		187.748		1	.000	3.185	2.699		3.759	
Childhood trauma events	-.049	.0235		-.095		-.003		4.396		1	.036	.952	.909		.997	
Suicidal tendencies	-.081	.0485		-.176		.014		2.788		1	.095	.922	.839		1.014	
Psychiatric comorbidities	-.105	.0297		-.163		-.047		12.479		1	.000	.900	.850		.954	
Prior treatment for depression	-.232	.0891		-.406		-.057		6.760		1	.009	.793	.666		.945	
